# Supplementary material for: Getting More Out of Biomedical Documents with GATE's Full Lifecycle Open Source Text Analytics
Source: PLoS Comput Biol. 2013 Feb 7;9(2):e1002854. doi: 10.1371/journal.pcbi.1002854 (PMC3567135; doi:10.1371/journal.pcbi.1002854)
Supplement: Dataset S1 — GATE software. Dataset S1 bundled with this paper contains a distribution of GATE (or see http://gate.ac.uk/download/). (TGZ) [file pcbi.1002854.s001.tgz › plos-gate/plugins/Tagger_MetaMap/doc/info/olteanu/utils/class-use/TextNormalizer.Java5Normalizer.html]

Uses of Class info.olteanu.utils.TextNormalizer.Java5Normalizer


---


|  |  |  |
| --- | --- | --- |
| **Overview** \| **Package** \| **Class** \| **Use** \| **Tree** \| **Deprecated** \| **Index** \| **Help** | |  |
| PREV   NEXT | **FRAMES**    **NO FRAMES**     **All Classes** |  |


---


## **Uses of Class info.olteanu.utils.TextNormalizer.Java5Normalizer**

No usage of info.olteanu.utils.TextNormalizer.Java5Normalizer

---


|  |  |  |
| --- | --- | --- |
| **Overview** \| **Package** \| **Class** \| **Use** \| **Tree** \| **Deprecated** \| **Index** \| **Help** | |  |
| PREV   NEXT | **FRAMES**    **NO FRAMES**     **All Classes** |  |


---
